# Association of breast carcinoma growth with a non-canonical axis of IFNγ/IDO1/TSP1

**DOI:** 10.18632/oncotarget.18781

**Published:** 2017-06-28

**Authors:** Bruno Lopes-Bastos, Liang Jin, Fiona Ruge, Sioned Owen, Andrew Sanders, Christopher Cogle, John Chester, Wen G. Jiang, Jun Cai

**Affiliations:** ^1^ Cardiff China Medical Research Collaborative, School of Medicine, Cardiff University, Cardiff CF14 4XN, UK; ^2^ School of Medicine, University of Florida, Gainesville, Florida 32610-0278, USA; ^3^ Division of Cancer & Genetics, School of Medicine, Cardiff University, Cardiff CF14 4XN, UK

**Keywords:** IFNγ, IDO1, TSP1, endothelial cells, breast invasive ductal carcinoma

## Abstract

Reciprocal interactions between cancers and the surrounding microenvironment have an important role in tumour evolution. In this study, our data suggested that through thrombospondin 1 (TSP1), tumour-associated microvessel provides a dormant niche to sustain inactive status of breast invasive ductal carcinoma (IDC) cells. TSP1 levels in the tumour stroma were negatively correlated with vascular indoleamine 2,3-dioxygenase 1 (IDO1) in IDC tissues. IDO1 is an intracellular enzyme initiating the first and rate-limited step of tryptophan breakdown. Lower stromal TSP1 levels and positive tumour vascular IDO1 staining seems to associate with poor survive of patients with IDC. IDC cells induced a significantly increase in IDO1 expression in endothelial cells (ECs). IFNγ exerts a similar effect on ECs. We hypothesized a tryptophan starvation theory that since tryptophan is essential for the synthesis of TSP1, IDO1 induce a decrease in tryptophan availability and a reduction in TSP1 synthesis in ECs, leading to overcoming the dormancy state of IDC cells and exacerbating conditions such as tumour invasion and metastasis. These findings identify a non-canonical role of IFNγ/IDO1/TSP1 axis in microvascular niche-dominated dormancy of breast invasive ductal carcinoma with a solid foundation for further investigation of therapeutic and prognostic relevance.

## INTRODUCTION

Invasive ductal carcinoma (IDC) is an aggressive form of breast cancer and the most common cancer in women worldwide [1]. Despite numerous advances in diagnosis and treatment, invasion and metastasis remain the primary cause of death associated with IDC. 10% to 30% of patients with IDC diagnosed at an early stage with no evidence of metastatic lymph node manifest with metastasis after >10 years of the first diagnosis. Within tumour microenvironment, interactions between tumour cells and stromal cells (such as tumour vascular endothelial cells) determine the extent of tumour growth [2]. While most tumour cells are detected and eliminated by host defence system, some stay in a dormant state where an equilibrium with the host system is reached-tumour dormancy [3, 4]. IDC recurrence probably is mainly due to tumour dormancy rather than the re-growth of the residual cancers in patients [5, 6, 7].

Prolonging tumour dormancy has been proposed as a promising approach to inhibit tumour growth and metastasis [8]. However, tumour dormancy is difficult to study, especially in clinical settings. In a metastatic mouse model, dormant IDC cells were observed near the lung, brain and bone marrow microvasculature [9]. Stable microvascular niche can sustain the quiescence of IDC cells. Thrombospondin 1 (TSP1), a large matrix glycoprotein, is the most abundant potent endogenous inhibitory component in the stable microvascular niche via mediating cell-to-cell and cell-to-matrix interactions. Lower levels of TSP1 expression frequently observed at the advance front of invasive breast cancer are significantly correlated with metastasis in tumour progression [10]. Vascular endothelial cells (ECs) are responsible for the majority of TSP1 secreted into the tumour stroma [11].

Indoleamine 2,3-dioxygenase 1 (IDO1) overexpresses in variety types of tumours including breast cancer [12, 13]. The pathologic significance of IDO1 in breast cancer involves in a complex of regulatory interactions of metabolism and immune. IDO1 is an intracellular immune checkpoint enzyme that catalyses tryptophan during the first and rate-limited step of L-tryptophan degradation, exerts a crucial immune tolerance role in inflammation response. There are conflicting results regarding the correlation of the high levels of IDO1 expression with lymph node involvement of breast cancer. However, the increased IDO1 in tumour endothelial cells was associated with limitation of the tryptophan influx from the blood circulation [14].

Interferon-γ (IFNγ) has been shown to induce IDO1 in a variety of malignancies [15]. A detailed study revealed that local IFNγ could directly target tumour vascular endothelial cells and affect tumour stroma [16]. IFNγ is a cytokine whose biological activity associated with modulating innate and adaptive immune responses [17]. There are conflict results regarding the role of IFNγ in tumour progression. Early *in vivo* study showed that neutralising IFNγ enhanced tumour growth, suggesting that IFNγ protects malignant lesions [18]. Consistently, the overexpression of dominant negative IFNγ receptors caused a significant increase in tumour growth *in vivo* models [19]. Surprisingly, there are reports that IFNγ protecting cells from inflammation insults might allow malignant cells to evade elimination, manifesting pro-tumorigenic activities [20]. For instance, intratumoral expression of IFNγ strengthened the aggressiveness of melanoma including lung colonization [21].

Since IDC represent one of the least immunogenic tumours, we hypothesized that IDC cells initiate a negative feedback loop in TSP1-dependent tumour growth arrest. Firstly, high levels of TSP1 in stroma suppressed the IDC cells directly. The antitumor effects result in recognition and elimination of the stromal TSP1 by the IDC cells, triggering an increase in IFNγ-stimulated IDO1 levels in the adjacent vascular ECs. Secondly, the vascular IDO1 serves as the major negative regulator of the stromal TSP1 proteins by degrading tryptophan. Thirdly, since sequencing analysis reveals that TSP1 contains a higher percentage of mannosylated L-tryptophan in the type 1 repeats [22], the resultant tryptophan deprivation leads to a reduction in IDC-associated vascular ECs synthesizing TSP1. Finally, a reduction in the stromal TSP1 proteins ultimately leads to the escape of the IDC cells from the siege of stromal TSP1. This study provided evidence that different signal regulation of crosstalk within tumour microenvironment might be a critical event in tumour dormancy regarding IDC development and metastasis. To this end, our observations not only confirm the notion of targeting IDO1 for breast cancer treatment but may raise potential concerns regarding the efficacy and safety of IFNγ for broadly clinical usage.

## RESULTS

### Endothelial cells exerts an inhibitory effect on IDC cells, leading to the cell cycle arrest of IDC cells

**Figure 1:**
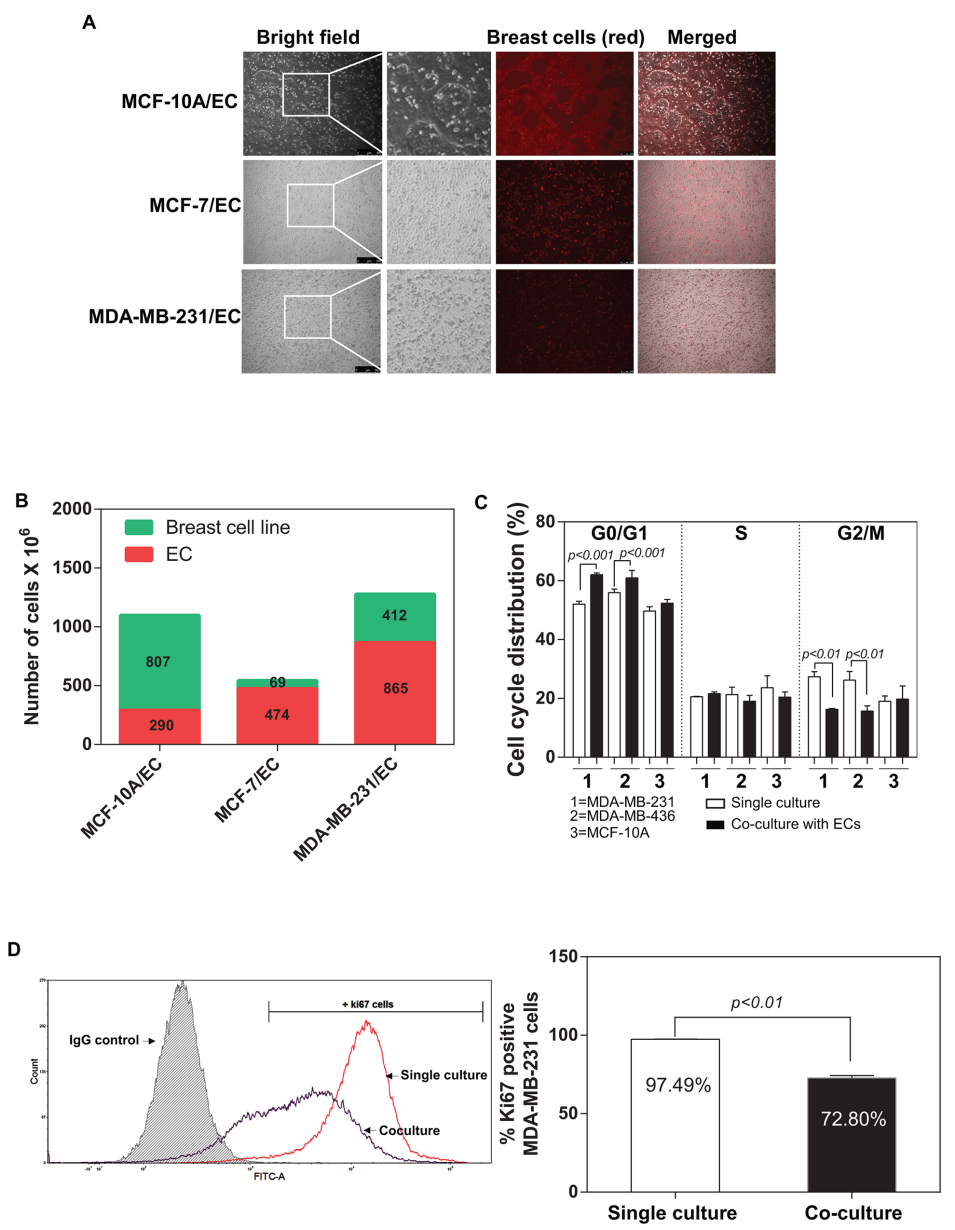
Co-culture of breast tumour cells with endothelial cells **(A)** Fluorescence microscope representative pictures of 2D co-culture *in vitro* model composed with different breast tumour cells and endothelial cells (ECs) in a 1:1 ratio. **(B)** Quantification of the ratio of the cells in co-culture after 48 hours by flow cytometry (1,000,000 cells of each cell type were initially seeded in each well). **(C)** Percentage of MDA-MB-231, MDA-MB-436 and MCF-10A cells in G0/G1, S and G2/M phase 48 hours after single culture or in co-culture with human dermal microvascular EC (HMEVCa-D) cells. **(D)** Sorting plots and gates were used for Ki67 analysis. The plot in red showed the percentage of Ki67 positive cells when MDA-MB-231 cells were cultured alone. The plot blue showed the percentage of Ki67 positive cells when MDA-MB-231 cells were co-cultured with HMEVCa-D for 48 hours.

Ghajar et al. 2013 showed that mutual interaction between breast tumour and endothelial cells within the tumour microenvironment might directly regulate the dormant disseminated tumour cells [9]. They demonstrated that endothelial cells-dominated vascular niche is a critical component in the dormancy of these cells. We developed an *in vitro* co-culture system and investigated the influence of ECs on the behaviours of breast tumour cells (described in MATERIALS and METHODS) (Figure 1A). Interestingly, the proliferation of a non-tumorigenic breast cell line (MCF-10A) was not affected by ECs and accounted for almost 80% of the total cell number after 48 hours (Figure 1B). In contrast, a substantial decrease in the percentage of breast tumour cancer cells was detected in both MCF-7 and MDA-MB-231 (13.1% and 30.6% respectively) (Figure 1B). We examined the effects of ECs in the induction of dormancy in IDC cells through flow cytometric analysis of the cell cycle of the MDA-MB-231, MDA-MB-436 and MCF-10A. Notably, the both MDA-MB-231 and MDA-MB-436 cells co-cultured with ECs had a high percentage of cells in the G0/G1 phase and a significantly low level in the G2/M phase in compared with control (single culture group), whereas MCF-10A did not exhibit a difference between co-culture and single culture (Figure 1C). Interestingly, ECs did not induce a detectable change in S phase. We further investigated whether ECs regulate proliferation of IDC cells by measuring a proliferation marker Ki67 using flow cytometry. Our data indicate that the interaction with ECs led to a significantly decrease in Ki67 positive MDA-MB-231 cells (Figure 1D). MDA-MB-231 cells are less active when co-cultured with ECs (Ki67+ MDA-MB-231 cells: 72.80%) than the single culture of MDA-MB-231 (Ki67+ MDA-MB-231 cells: 97.49%). However, flow cytometric analysis did not show any difference in senescence marker p21 expression in MDA-MB-231 cells between single culture or co-culture with ECs (Supplementary Table 1 and Supplementary Figure 1A).

### EC-derived TSP1 suppresses proliferation of IDC cells

**Figure 2:**
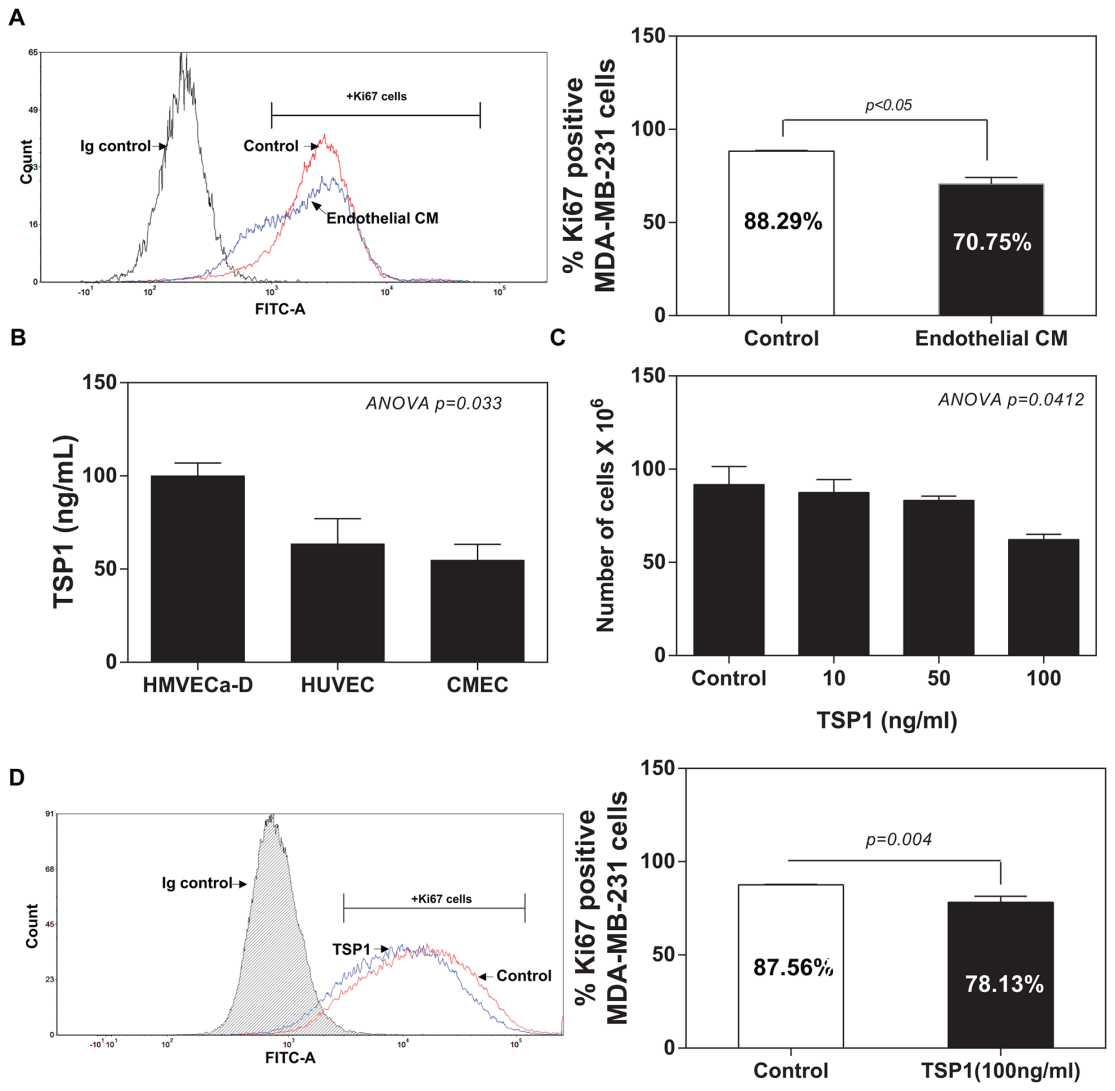
TSP1 reduces proliferation of IDC cells **(A)** Comparison of the percentage of Ki67 positive MDA-MB-231 cells cultured with the HMEVCa-D cell-conditioned medium with the normal control groups. **(B)** ELISA analysis of TSP1 expression levels in the supernatants obtained from a panel of vascular endothelial cells. **(C)** Cell viability assay showing that recombinant TSP1 proteins inhibit proliferation of MDA-MB-231 cells with the greatest effect at 100ng/ml. **(D)** Decreased percentage of Ki67 positive MDA-MB-231 cells after treated with 100ng/ml of TSP1 for 24 hours.

In a recapitulation of the findings from *in vitro* co-culture of IDC cells with ECs, we co-cultured MDA-MB-231 cells with conditioned medium from ECs. As shown in Figure 2A, flow cytometric analysis revealed that the treatment of conditioned medium from ECs for 48 hours induced a significant reduction in the proliferation of MDA-MB-231 cells (Ki67+MDA-MB-231 cells: 70.25%) compared to the normal medium group (Ki67+ MDA-MB-231 cells: 88.29%). We speculated that vascular ECs within tumour microenvironment might suppress the proliferation of breast cancer cells via the secreted molecules, such as TSP1. We also analysed three standard endothelial cell (EC) lines for the expression of TSP1 proteins, including human dermal microvascular endothelial cells (HMVECa-D), human vascular umbilical cord ECs (HUVECs) and a cerebral microvascular ECs (CMECs). ELISA failed to detect any levels of TSP1 from the EC medium (data not shown). ELISA analysis showed detectable TSP1 proteins ranged from 100 to 50 ng/ml from the EC-conditioned medium from the all three tested ECs (Figure 2B). To determine whether EC-derived TSP1 suppresses IDC cell viability, we treated MDA-MB-231 cells with recombinant human TSP1 proteins at different concentrations (0, 10, 50 and 100 ng/ml) for 48 hours. TSP1 treatment did exert a significantly inhibitory effect on the viability of MDA-MB-231 cells at dose-dependent manner (Figure 2C). Flow cytometric analysis of Ki67 expression confirmed that TSP treatment (100 ng/ml) significantly reduced Ki67+ MDA-MB-231 cells compared with control group (78.13%±3.250 vs. 87.56%±0.2881; 95%CI -18.48 to -0.3843; R2=0.6768; p=0.0444) (Figure 2D). Since TSP1 suppressed the proliferation of MDA-MB-231 cells, it was logic for us to assess whether TSP1 treatment also induces apoptosis of MDA-MB-231 cells. Surprisingly, TSP1 treatment (100 ng/ml) did not cause a detectable apoptotic effect on MDA-MB-231 cells compared to the control group (Supplementary Figure 1B).

### Differential expressions of TSP1 in IDC tissues with its stromal expression inversely are related to IDC progression

**Figure 3:**
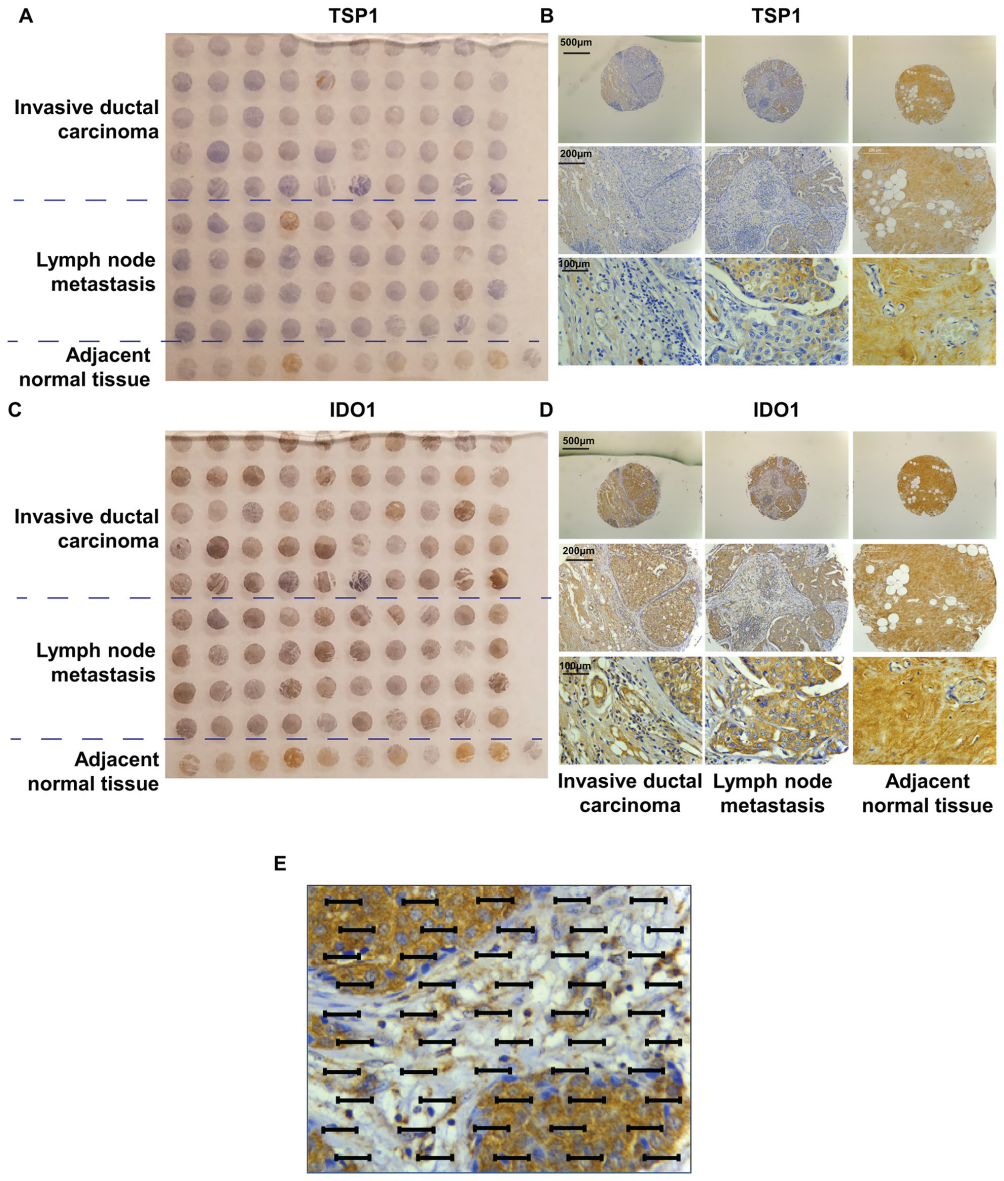
TMA of human invasive ductal carcinoma patients **(A)** Photograph of IDC patient tissue microarry immun-ohistochemically stained for TSP1. **(B)** Representative immunostaining of TSP1 in a tumour TMA compiled from adjacent normal tissue, lymph node metastasis and primary invasive ductal carcinoma. **(C)** Photograph of IDC patient tissue microarray immunohistochemically stained for IDO1. **(D)** Representative immunostaining of IDO1 in a tumour TMA compiled from adjacent normal tissue, lymph node metastasis and primary invasive ductal carcinoma. **(E)** An eyepiece systemic point-sampling grid with 100 points and 50 lines to count the number of points overlying positively-stained structures at 400× magnification for histomorphometric analysis.

We performed semi-quantitative immunohistochemical (IHC) analysis of the expressions of TSP1 in commercial tissue microarrays of 100 human breast cancer specimens or adjacent normal tissues, scoring expression from 0 (no expression) to 3 (high expression). We observed that TSP1 immunostaining was very weak in invasive breast carcinoma and an increase in TSP1 staining in adjacent normal tissues (Figure 3A, 3B). Interestingly, there was an increase in TSP1 expression in metastasis to lymph nodes. TSP1 was observed as typical fine fibrillary stromal staining as well as in the basement membrane of duct space (Figure 3B). TSP1 expression was higher in adjacent normal tissue than in invasive breast carcinoma (staining intensities, SI: 1.100±0.100 vs. 0.5200±0.08685, p<0.0001, Table 1), but higher in metastatic lymph node than in adjacent normal tissues (SI: 1.226±0.1006 vs. 1.100±0.100, p<0.0001, Table 1).

**Table 1:** Summary of IHC intensity results

		Staining intensity (SI)				Average ±SEM
		0	1	2	3	
Adjacent normal (n=10)	TSP1	0	9	1	0	1.100±0.100
	IDO1	7	2	1	0	0.6000±0.2667
Invasive ductal carcinoma (n=50)	TSP1	26	23	0	1	0.5200±0.08685***
	IDO1	0	23	23	4	1.560±0.09545***
Lymph node metastasis (n=40)	TSP1	9	26	3	2	1.226±0.1006***
	IDO1	0	26	13	1	1.375±0.08539***

Statistical significance was determined by One-way ANOVA. ***P<0.0001

**Figure 4:**
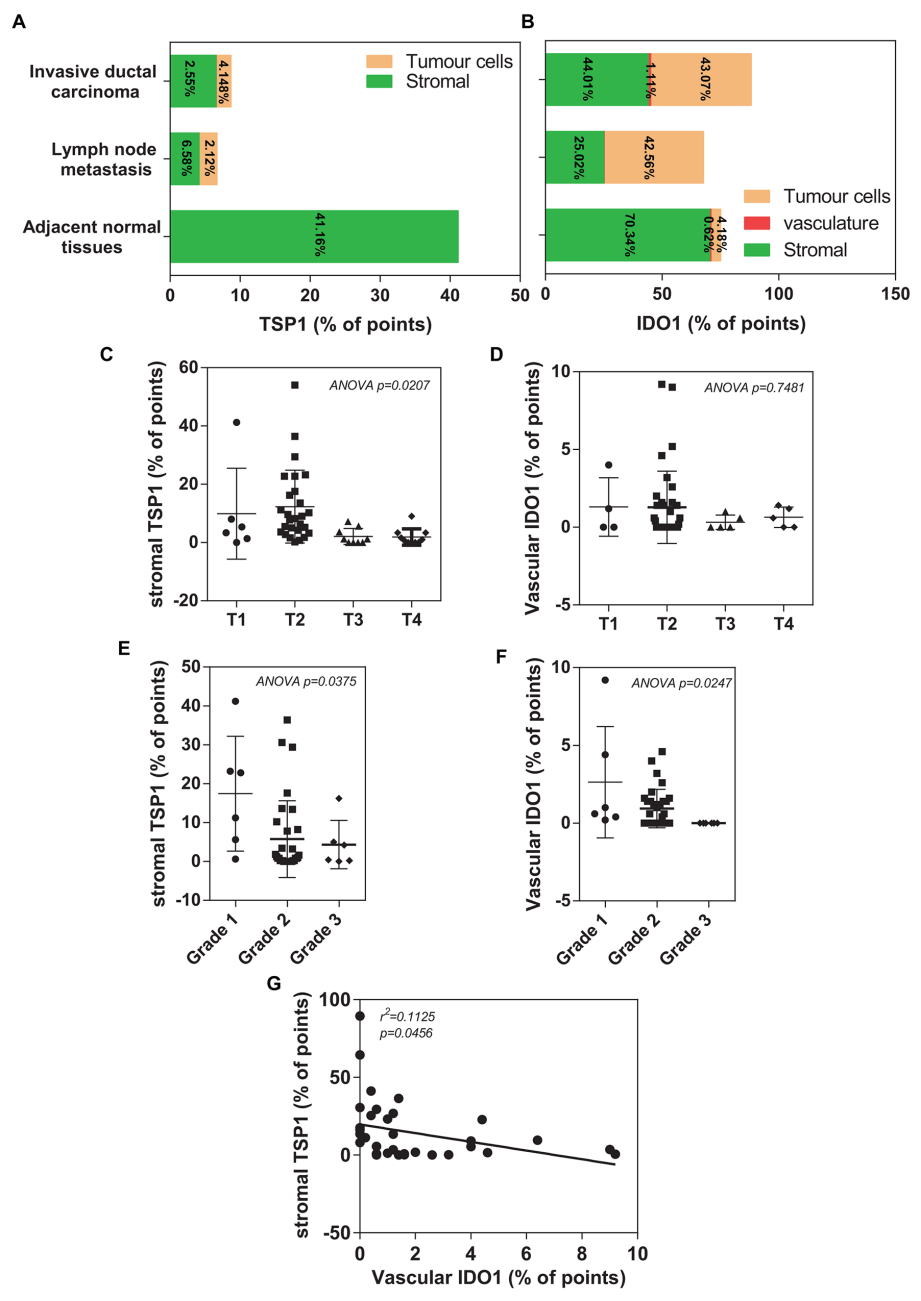
Histomorphometric analysis of TSP1 and IDO1 in a TMA of human invasive ductal carcinoma patients **(A)** Plot of percentage of immunostaining TSP1 covering different areas on tissue microarray, including adjacent normal tissues, invasive ductal carcinoma and lymph node metastasis. **(B)** Plot of percentage of immunostaining IDO1 covering different areas on tissue microarray, including adjacent normal tissues, invasive ductal carcinoma and lymph node metastasis. **(C)** A significantly association between decreased histomorphometric scores of the stromal TSP1 and high TNM status (p=0.0207). **(D)** A trend toward reduced histomorphometric scores of the vascular IDO1 was observed in patients with IDC who had an overall poor outlook (T3 and T4), compared with those patients with a relative good prognosis (T1 and T2). **(E)** Decreased histomorphometric scores of the stromal TSP1 was significantly correlated with poor differentiated grade tumours (p=0.0375). **(F)** Histomorphometric scores of the vascular IDO1 significantly decreased in IDC tissues with poor differentiated grade with the observation that the lower vascular IDO1 were in high TNM status (p=0.0247). **(G)** Plot of the vascular IDO1 (histomorphometric scores as % of points) on the horizontal axis versus the stromal TSP1 (histomorphometric scores as % of points) on the vertical axis. The trend in the points is given by the line with as statistical significance (R^2^=0.1125, p=0.0456).

To further measure the positive staining area of the commercial tissue microarrays of 100 human breast cancer specimens or adjacent normal tissues, we performed the histomorphometry analysis by adapting an eyepiece systematic point-sampling technique (described in *MATERIALS and METHODS*) (Figure 3E). TSP1 staining occupied the most of the stroma (Figure 4A). Stromal TSP1 was seen 40 out of 50 primary IDC cases (Table 2). As shown in Figure 4C, ANOVA analysis showed that there was a significant and inverse correlation between the degree of tumour stromal TSP1 and the TNM grading (R^2^=0.1823; p<0.05), even though mean TSP1 values in the T2 seemed to increase compared to T1 group. The T1 group (n=6) represent patients with tumour invading submucosa (mean TSP1: 9.90%). The T2 (n=27) contains patients with tumour invading muscularis propria (mean TSP1: 12.31%). The T3 patients (n=9) had tumours invading through muscularis propria into subserosa or non-peritonealized pericolic or perirectal tissues (mean TSP1: 2.089%), whereas T4 group (n=10) containing tumour directly invades other organs or structures and/or perforate visceral peritoneum (mean TSP1: 1.900%). Another tool for the evaluation of tumour prognosis is to evaluate the differentiation of the tumour cells (Supplementary Table 1). A higher grade usually corresponds to a more aggressive cancer (Figure 4E). There is an even more apparent difference (R2=0.1480; p<0.05) in the degree of tumour stromal TSP1 between the well differentiated Grade 1 tumours (mean TSP1: 17.43%; n=6), than the modestly differentiated Grade 2 classed tumours (mean TSP1: 5.738%; n=32), and the poorly differentiated Grade 3 tumours (mean TSP1: 4.333%; n=6).

**Table 2:** Summary of IHC morphometric results

		Mean	minimum	maximum
Tumour Cells (%)	TSP1	2.12	0.00	16.50
	IDO1	43.068	5.00	98.60
Stromal (%)	TSP1	6.576	0.00	42.20
	IDO1	44.012	0.00	76.60
Vasculature (%)	TSP1	0.016	0.00	0.08
	IDO1	1.112	0.00	4.60

The units “% of points” indicate the number of points overlying the structure of interest divide by total number of points overlying the tissues.

### Stromal TSP1 expression is inversely related to vascular IDO1 expression in primary IDC tissues

Recent studies suggested that IFNγ can induce an overexpression of IDO1 in varieties types of tumours including breast cancer, followed by a breakdown of tryptophan [23]. We also performed IHC staining of IDO1 in the same commercial tissue microarrays as described previous (Figure 3C, 3D). In contrast to TSP1 expression, adjacent normal tissues expressed relatively small amounts of IDO1 (SI of 0.60±0.2667), compared to IDC (SI of 1.560±0.09545, p<0.001) (Figure 3D and Table 1). Again, the IDO1 expression pattern in metastatic lymph nodes was different, with the intensity of IDO1 staining in lymph node metastasis lower than in primary IDC, with average SI of 1.375±0.08539 (p<0.001).

We found that none of IDC tissues were entirely negative for IDO1 staining, many of them also were in stromal fraction (Figure 4B). However, vascular IDO1 staining was present in most of primary IDC cases (49/50) (Table 2). Unfortunately, no significant association is found between the vascular IDO1 and TNM grading (R^2^=0.04933; p>0.05) (Figure 4D). Interestingly, the vascular IDO1 expression is significantly correlated with the differentiation grade of IDC. As shown in Figure 4F, the differentiated degree of primary invasive well-differentiated Grade I tumours contain 2.633% of vascular IDO1, which is reduced to 0.9438% of vascular IDO1 in the modestly differentiated Grade 2 and disappeared at the poorly differentiated Grade 3.

Since TSP1 contain a high percentage of tryptophan, [22] we speculated that the vascular IDO1 acts as a critical negative regulator of the tumour suppressive activity of stromal TSP1 in human breast cancer. A good correlation of IHC staining between the stromal TSP1 and vascular IDO1 was observed in primary IDC tissues. An increase in the vascular IDO1 was significantly associated with a decrease in the stromal TSP1 in IDC tissues (Figure 4G), suggesting that low stromal TSP1 and high vascular IDO1 expressions at the primary site may be considered as markers for the evolution of the cancerous breast lesions.

Furthermore, overall survival was compared (between low vs. medium/high groups) for both histomorphometric scores of the stromal TSP1 and the vascular IDO1. Unfortunately, there was no statistically significant difference between the stromal TSP1-low and stromal TSP1-medium/high groups (HR: 1.781 vs. 0.5613; 95%CI: 0.5192 to 6.113 vs. 0.1636 to 1.926; p=0.3587) (Supplemental Figure 2A). However, total survival trended toward superiority in the stromal TSP1-medium/high group. For the vascular IDO1, there was no statistically significant different between the low and medium/high group (HR: 0.8514 vs. 1.188; 95% CI: 0.2675 to 2.647 vs. 0.3778 to 3.738; p=0.8714). Interestingly, the total survival curve also exhibited a trend of superiority for the vascular IDO1-low group compared to the vascular IDO1-medium/high group (Supplementary Figure 2B).

### IDC cell-derived IFNγ abolishes the inhibitory effect of stromal TSP1 on the IDC cells

**Figure 5:**
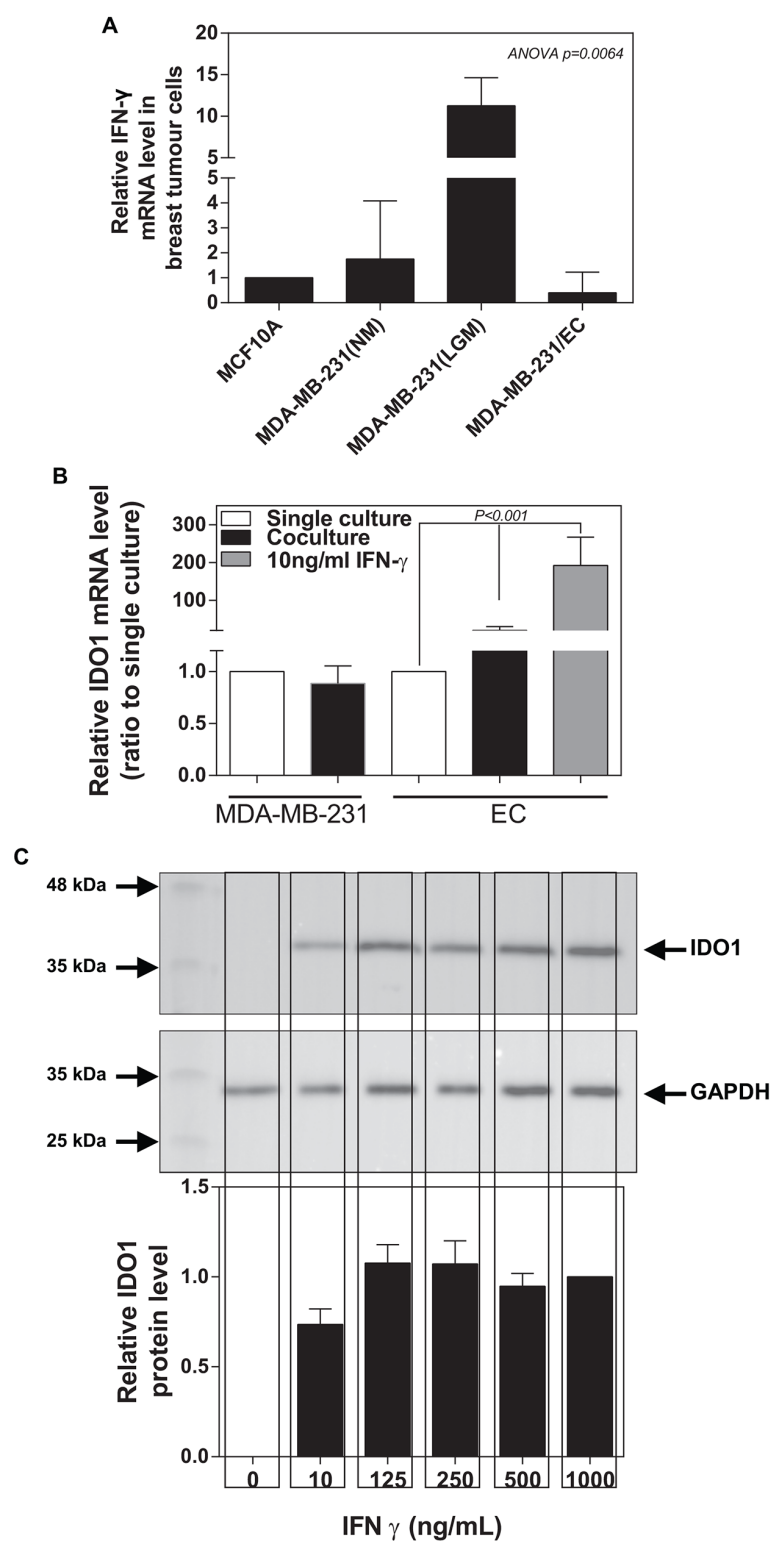
Breast tumour cancer cells express IFNγ inducing an increase in IDO1 expression in ECs **(A)** IFNγ mRNA expression is significantly up-regulated in MDA-MB-231 than those in MCF-10A (normal breast epithelial cells), which is further enhanced by low glucose (LGM) exposure for 48 hours compared with normal glucose (NM) medium. Also, IFNγ expression in MDA-MB-231 is reduced after co-cultured with endothelial cells for 48 hours. **(B)** Differential IDO1 mRNA expression in MDA-MB-231 and ECs occurs after single culture or co-culture for 48 hours. IDO1 mRNA levels significantly increase in endothelial cells after treated with IFNγ for 48 hours. **(C)** Western blot analysis confirms that IFNγ treatment induces an increase in IDO1 protein levels in endothelial cells using anti-IDO1 antibody (the upper panel), analysed by measuring band density (the low panel). GAPDH was used as an internal control.

IFNγ is predominately produced by several immune cells as part of the innate immune response. However, a previous study revealed that different immunoreaction to IFNγ in three breast lesions (benign, in situ and infiltrating breast cancers) with the highest level in the in situ lesion [16]. Thus, we speculate that breast cancer cells might also express IFNγ. We detected that MDA-MB-231 (tumorigenic metastatic breast cancer line) expressed a higher mRNA level of IFNγ than MCF-10A (non-tumorigenic breast tumour cell line) (Figure 5A). We also examined whether low glucose medium (LGM) could further increase IFNγ expression in IDC cells. Indeed, we found that low glucose medium (1 mM glucose) caused an almost 100-fold increase in IFNγ expression in MDA-MB-231 cells, which was completely abolished by the co-cultured with ECs (Figure 5A). Meantime, we observed that ECs significantly increased IDO1 expression in the presence of MDA-MB-231 cells or 10 ng/ml IFNγ, while ECs seems not to affect IDO1 expression in MDA-MB-231. (Figure 5B). Western blot data further confirmed that IFNγ induced an increase in IDO1 expression in the ECs at dose-dependent manner (Figure 5C).

**Figure 6:**
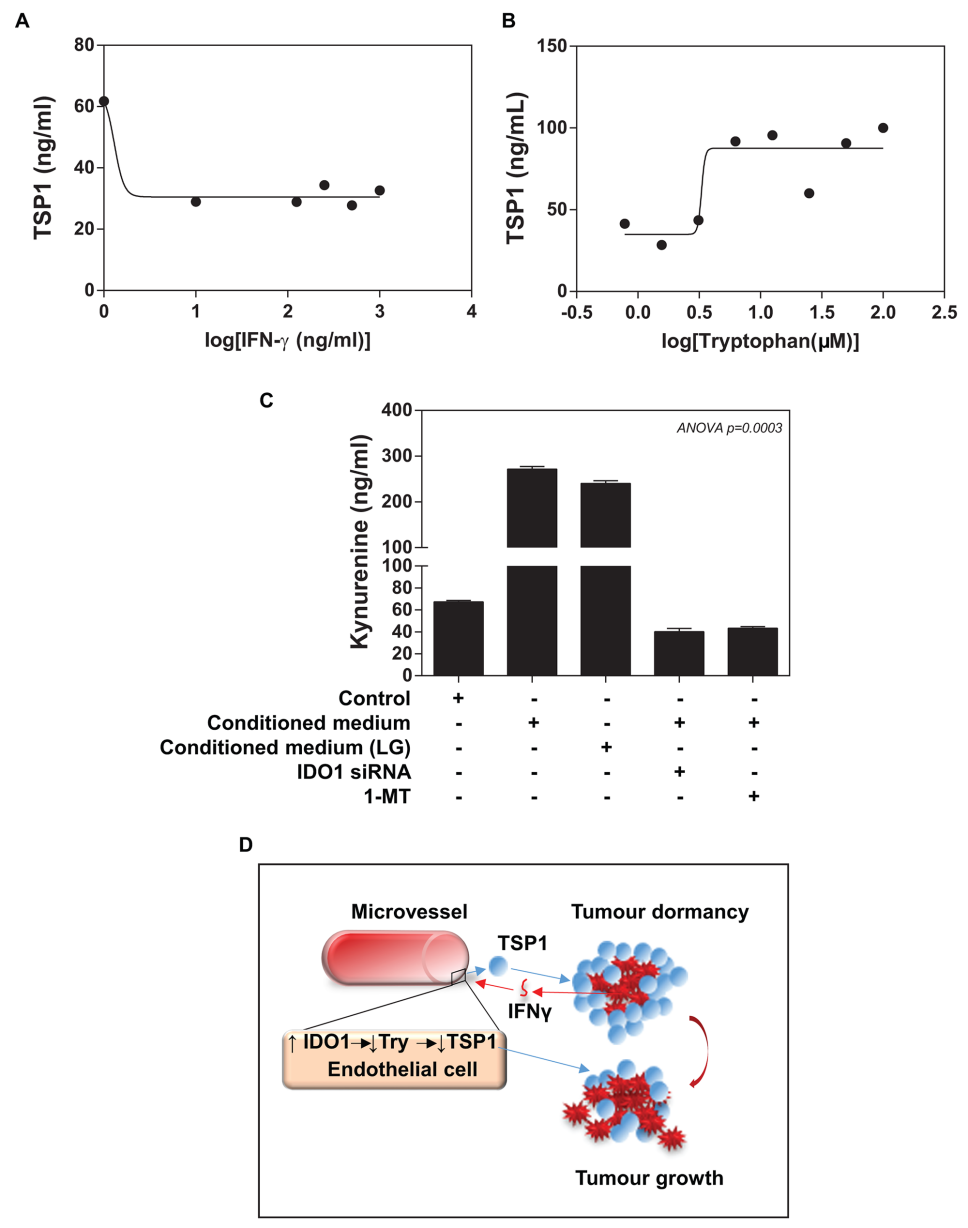
Functional importance of IFNγ/IDO1/TSP1 axis **(A)** ELISA assay shows that IFNγ induces a reduction in HMEVCa-D cells secreting TSP1 into extracellular space with IC_50_=10ng/ml. **(B)** HMEVCa-D cells were treated with the medium implemented with different concentrations of tryptophan. Addition of tryptophan for 72 hours resulted in an increase in TSP1 expression in ECs with IE_50_ of approximately 5μM. **(C)** ELISA assay reveals that the MDA-MB-231 cell-conditioned medium (normal or low glucose) induced a significantly increase in intracellular L-kynurenine protein levels in HMEVCa-D cells, an indication of tryptophan degradation, which is reversed by IDO1 siRNA and IDO1 inhibitor-1-Methyl-tryptophan (1MT). **(D)** A possible non-canonical role of IFNγ/IDO1/TSP1 axis in microvascular niche-dominated tumour dormancy of breast invasive ductal carcinoma cells. High levels of TSP1 in stroma suppressed the IDC cells directly. The antitumor effects result in recognition and elimination of the stromal TSP1 by the IDC cells, triggering an increase in IFNγ-stimulated IDO1 levels in the adjacent vascular ECs. The vascular IDO1 serves as the major negative regulator of the stromal TSP1 proteins by degrading tryptophan, one essential amino acid for TSP1 synthesis, which ultimately leads to a reduction in the stromal TSP1 proteins and the escape of the IDC cells from siege of stromal TSP1.

Recently, Ghaiar et al. attributed the breast tumour dormancy to high concentrations of TSP1 in a microenvironment, which enabled breast cancer cell to remain quiescent [9]. We assessed whether there is a change in TSP1 levels of ECs upon treatment of different concentrations IFNγ. Indeed, ELISA analysis revealed that IFNγ treatment caused a dose-dependent inhibition of TSP1 expression in ECs (IC_50_ ≈10 ng/ml of IFNγ) (Figure 6A). Since TSP1 contains a high percentage of tryptophan, we determined whether the availability of extracellular tryptophan could affect the TSP1 synthesis in ECs. We loaded ECs with the medium implemented with different concentrations of tryptophan. Our ELISA data showed that addition of tryptophan for 72 hours resulted in a dose-dependent increase in TSP1 expression in ECs (IE_50_ ≈5μM of tryptophan) (Figure 6B and Supplementary Figure 3A).

### IFNγ-induced vascular IDO1 deprives the availability of tryptophan for TSP1 synthesis by ECs

Addition to the immunomodulatory effects of IDO1 on tumour cells, we speculated that IDC cells (via IFNγ) -induced IDO1 in ECs could catalyse the oxidation of L-tryptophan into kynurenine, which results in a reduction in the availability of tryptophan for the TSP1 synthesis by ECs. Accordingly, we measured the L-kynurenine levels of ECs as a way to assess IDO1 enzymatic activity. We, indeed, observed a marked increase in the extracellular L-kynurenine of ECs treated with the conditioned medium from MAD-MB-231 cells (normal glucose and low glucose), indicating a significant increase in tryptophan degradation (Figure 6C). Apart from IDO1, there are two other enzymes responsible for catalysing tryptophan including tryptophan 2,3-dioxygenase (TDO) and IDO2. We used a commercial IDO1 siRNA to successfully knockdown IDO1 expression up to ∼90% in ECs pretreated with the conditioned medium of MDA-MB-231 (Supplementary Figure 3B), while TDO and IDO2 mRNA were not affected (Supplementary Figure 3C). Interestingly, the conditioned medium from MDA-MB-231 cells did not increase either TDO or IDO2 expression in ECs, and our IDO1 siRNA treatment did not lead to the detectable upregulation of both enzymes (Data no shown). To further confirm IDO1-catalysed tryptophan as the primary reason for the TSP1 reduction, we measured L-kynurenine levels of ECs upon IDO1 siRNA treatment. IDO1 siRNA treatment significantly reduced the L-kynurenine levels of ECs treated with either co-cultured with the conditioned medium or LGM of MDA-MB-231 (Figure 6C). Furthermore, 1-methyl-[D]-tryptophan (1-MT), an IDO1 inhibitor, caused a remarked reduction in L-kynurenine levels of ECs (Figure 6C). Our data provide clear evidence that the elevated endothelial IDO1 caused a decrease in intracellular tryptophan levels. These experiments provide initial evidence that IDC cells, in part via IFNγ, induce endogenous IDO1 expression by ECs serves as an evasive mechanism against TSP1 of tumour dormancy (Figure 6D).

## DISCUSSION

TSP1 is a large matricellular glycoprotein in tumour stroma. Like other matricellular proteins, TSP1 exerts a multiple functions, even sometimes opposite, on tumour progression depending on the molecular nature of malignant lesions [24, 25]. Many non-platelet sources have been identified to produce TSP1 within the tumour microenvironment, such as endothelial cells [26]. cancer cells (adhesive or circulating) [27]. Ghajar et al. demonstrated that the quiescent breast tumour cells often enter into a long-term dormancy [9]. The fact that the dormant breast cancer cells in or near microvasculature led us to speculate that the vascular endothelial niche might play a direct role in the quiescent phenotype of these cells via secretion of TSP1 into the tumour microenvironment. Our tissue microarray analysis revealed that the stromal TSP1 expression is inversely correlated with the increased malignancy of invasive ductal carcinoma (IDC) with an indication of some survival benefit. *In vitro* experiments further demonstrated that the increased TSP1 concentration conferred the quiescent phenotypes of IDC cells. However, current reports regarding the effects of TSP1 on tumour progression are contradictory, with both negative and positive roles. For instance, TSP1 has been reported to promote progression of many other cancer types including glioma [28], melanoma [29], ovarían [30], and pancreatic carcinomas [31]. One plausible explanation is that TSP1 possesses multiple receptors, therefore, the multifaceted effects of TSP1 are due to the varied receptor expression profiles. Apparently, the strategy of simply direct targeting TSP1 without considering its pleiotropic effects for each case may induce severe unwanted side-effects as well as loss its beneficial functions. TSP1 contain a high percentage of tryptophan. Tryptophan is the rarest essential amino acid in humans, and its levels are regulated mainly by the balance between the tryptophan absorbance from blood as well as its degradation and its use in protein synthesis. Tryptophan degradation occurs mostly through the kynurenine pathway, in which indoleamine-2,3-dioxygenase (IDO or IDO1) and a splice variant of IDO1 known as IDO2 catalyse the oxidation of L-tryptophan into kynurenine with reducing the availability of tryptophan in the microenvironment and cells [32]. Tryptophan deprivation by IDO1 has been associated with tumour immune tolerance[33].

Several human cell types have been detected to express IDO1, such as activated dendritic cells, macrophages, endothelial cells, fibroblasts and multiple tumour cells [15, 34, 35]. In agreement with these statements, our *in vitro* experiments revealed that endothelial cells co-cultured with malignant breast cancer cells or IFNγ highly expressed IDO1. In a tumour tissue array, the intensity and area of vascular IDO1 staining positively correlated with the stage of breast cancer. Furthermore, low vascular IDO1 expression was found to have a trend of the superiority of survival. This finding will require further confirmation, however, is consistent with the clinical studies from the other groups showing that IDO1 is gradually up-regulated in a variety of cancer patients. High levels of its expression or enzyme activity in various cancer types closely associated with poor prognostic outcome [36–38]. For instance, IDO1-expressing colorectal cancer cells are more likely to metastasize to distant organs [39]. In breast cancer, high IDO1 expression correlated with lymph node involvement and with worse recurrence-free survival [23].

In this study, we demonstrate that ECs induced a decrease in proliferation of metastatic breast cancer cells with cell cycle arrest at the G0/G1 phase. We found that IDC cell cycle arrest might be due to quiescence, but no senescence. In the context of cellular dormancy, such quiescence is a better fit for the cell cycle arrest. Our co-culture system also showed that MDA-MB-231 cells are capable of inducing IDO1 expression in ECs. The conditioned medium from MDA-MB-231 cells showed the similar results, but not statistically significant (data not shown), which could be due to the lack of spatial and temporal parameters, demonstrating that the co-culture system adopted in this study is more closer to *in vivo* situations.

Our study confirmed that IFNγ induces the expression of IDO1 in ECs. The tumour cell plasticity enables the adjustment and changes the adverse microenvironment by influencing neighbouring stromal cells to involve the secretion of soluble factors. For instance, IFNγ has been shown to elevate in the tumour stroma [40]. IFNγ was initially identified to play a significant role in the detection and elimination of tumour cells as well as tumour surveillance by enhancing tumour cell immunogenicity [17]. The profound immunomodulatory effects of IFNγ has long been inspiring clinical applications in antitumour functions. However, the clinical development of IFNγ was mostly inconclusive due to many of these trials lacking efficacious effects of IFNγ. Although IFNγ exhibited antiproliferative, antiangiogenic and pro-apoptotic effects on cancer cells, there is growing reports of its pro-tumorigenic behaviours [41–43]. There are some extreme cases that the IFNγ-treated patients even fare worse than the untreated population [44–46]. Moreover, there are reports of IFNγ being pro-tumorigenic even though its inhibiting tumour growth was only evident at a much high dose [1].

Taken together, we for the first time provided the rationale for the non-canonical role of IFNγ in IDC cells evading from tumour dormancy (Figure 6D), other than immunomodulation. First, breast cancer cells, in part via IFNγ, induce IDO1 expression in endothelial cells, leading to tryptophan degradation. Second, the resultant reduction in the availability of tryptophan affects TSP1 synthesis and secretion, which tips between the production and degradation of TSP1 in favour of a decrease in TSP1 levels in the microenvironment. The deprivation of TSP1 exerts a permissive role in the breast cancer cells evading tumour dormancy. More importantly, our observations contribute significantly to our understanding of divergent signal-regulation of tumour dormancy as a critical event in IDC development and metastasis via crosstalk with tumour stroma. Further, these studies raise potential concerns regarding the efficacy and safety of IFNγ for the cancer treatment. In term of breast cancer, up to 30% of those diagnosed as in situ breast cancer exhibit tumour dormancy with metastasis-free [7], which will make these individuals particularly vulnerable to any adverse activities of IFNγ-related breast cancer management.

## MATERIALS AND METHODS

### Cell culture

A panel of human ECs includes human dermal microvascular EC (HEMVEca-D) (Lonza Biologies plc. Berkshire, UK), human umbilical vein EC (HUVEC) (ICLC, Genova, Italy) and human cerebral microvascular EC (CMEC) (Dr Yasuteru Sano, Yamaguchi University School of Medicine, Japan). A co-culture system was adopted to study the interaction between IDC and endothelial cells. ECs were stimulated by three types of breast tumour cells. MDA-MB-231 and MDA-MB-436 are human tumorigenic metastatic breast cancer cell lines, MCF-7 is a tumorigenic but non-metastatic breast cancer cell line while MCF-10A serves as a non-tumorigenic breast epithelial cell, respectively [47]. Brest tumour cells were treated with 1 μg/ml CellTrack™ Orange CMRA (Life Technologies, Life Technologies, Paisley, UK) in a serum-free basal medium at 37°C for 1 minutes. The breast cell suspensions added to the top of the HMEVCaD cell monolayer. After 48 hours, the co-cultures were trypsinized and resuspended in PBS. Breast tumour cells and ECs were isolated from the co-cultures through cell sorting. We used MoFlo™ XDP (Beckman Coulter (UK) Ltd., High Wycombe, UK) for cell sorting by the size and labelled fluorescent dyes (Supplementary Figure 2A). We tested single cell populations for sequential experiments.

Breast tumour cells were treated with the HMEVCa-D cell-conditioned medium, or TSP1 (Sigma-Aldrich Company Ltd., Dorset, UK) for the indicated times. In some experiences, MDA-MB-231 cells were also treated with low glucose medium (1 mM glucose). 25 μM of 1-Methyl-tryptophan (1-MT, Sigma-Aldrich Company Ltd., Dorset, UK) was used to treat HMEVCa-D cells. At this concentration, 1-MT is not cytotoxic to the cells (Supplementary Figure 3D). In some experiments, HMEVCa-D cells were subject to treatments of MDA-MB-231 cell-conditioned medium (normal or low glucose), or IFNγ or tryptophan (Sigma-Aldrich Company Ltd., Dorset, UK) for the indicated times.

### IHC analysis of tissue microarray (TMA)

We purchased a tissue microarray (TMA) slides of breast invasive ductal carcinomas (n=100) generated by US Biomax Inc. (Rockville, MD, USA). This tissue microarray contains forty six cases of invasive duct carcinomas, one neuroendocrine carcinoma, three medullary carcinomas, forty lymph node metastatic carcinomas, ten adjacent normal tissues (single core per case) (Figure 3A, 3C). IHC analysis was carried out with modified protocol described previously [48]. Prior to staining, the slides were dewaxed and hydrated. For antigen retrieval, slides were immersed in citrate buffer (pH 8.0) and heated in a microwave (≥700W) for 20 minutes. The slides were quenched with endogenous peroxidase by incubation with 3% H_2_O_2_ for 5 minutes and washed 3 times with TBS. A blocking buffer (1% BSA, 1% Marvel and 5% goat serum in TBS) treated slides containing serial cores from adjacent tissue sections for 1 hour to block any non-specific binding. Rabbit anti-TSP1 (1:400) or Rabbit anti-IDO1 (1:200) polyclonal antibodies (Abcam, Cambridge, UK) stained the slides at 4°C for overnight. We used the antiserum against primary antibody as the negative control. After primary antibody incubation, the slides were washed 3 times in TBS for 5 minutes and incubated with secondary antibody for 30 minutes at room temperature. The slides were then washed 3 times in TBS for 5 minutes each time and incubated with the ABC complex for 30 minutes (Vector Laboratories, Peterborough, UK). The colour reaction was developed with 3,3’-diaminobenzidine (DAB) and the sections were then counterstained with haematoxylin (Vector Laboratories, Peterborough, UK). Finally, sections were washed in tap water, dehydrated through a series of graded ethanol, cleared in xylene, and mounted in DPX, followed by observation and imaging under an optical microscope. At × 200 magnification, the staining intensity was assessed in different cell types as 0 (negative), 1 (weak), 2 (intermediate) and 3 (strong) by two observers (FR, LJ).

### Histomorphometric analysis of TMA

The area fraction of staining occupied by the tumour cells, stroma and vascular ECs was evaluated. We use an eyepiece systemic point-sampling grid with 100 points and 50 lines to count the number of points overlying positively-stained structures at 400× magnification (Figure 3E) as previously described [47]. Measurements were averaged over five microscopic fields to obtain an indexed percentage. Comparisons were performed in 20% of the staining by the two observers (LJ, JC), the coefficient of variation for the inter-observer error regarding cell count was <5%.

### Flow cytometric analysis

MDA-MB-231 cells (single cultured or co-cultured with HMEVCa-D cells for 48 hours) were subject to assess the proliferative status (Ki67 expression) using flow cytometry analysis. MDA-MB-231 cells were fixed in 4% formaldehyde for 10 minutes, followed by adding ice-cold methanol to gain a final concentration of 90% methanol for 30 minutes. The cells were labelled with rabbit anti-Ki67 polyclonal antibody conjugated with Alexa 488 or rabbit IgG isotype control-Alexa 488 (Cell Signalling Technology, Inc. MA, USA) at room temperature for 1 hour. Flow cytometric analysis was using BD FACSCANTO II (Beckman Coulter (UK) Ltd., High Wycombe, UK).

We analysed the cell cycle by quantification of DNA contents via DNA binding dyes. In S phase, cells have more DNA than those in G1 phase. The cells in G2 are approximately twice as bright as those in G1 phase. Breast tumour cells (single culture or co-culture with ECs for 48 hours) were fixed in 70% ethanol for 2 hours on ice. After PBS wash, 2ug/ml of propidium iodide (PI) was added to the cells for 30 minutes at room temperature. We performed flow cytometric analysed in BD FACSCANTO II (Beckman Coulter (UK) Ltd., High Wycombe, UK). The forward scatter (FS) and side scatter (SS) were used to select single cell population. The PI histogram plot revealed the percentage of cells in each cell cycle phase according the manufacturer’s instructions.

### Viability assay

A viability assay called crystal violet assay was performed as previously described [47]. Briefly, 100μl cells were incubated in each well of 96-well plates at 1×10^5^ cell/ml. The cells were fixed in 4% paraformaldehyde in PBS for 15 minutes. After being washing with H_2_O, the plates were stained with 0.1% crystal violet solution for 20 minutes. The plates were washed with H_2_O and allowed to be air dry, followed by adding 100μl 33% of acetic acid to each well. Absorbance of the staining was measured by an automatic microtitre plate reader at 590nm.

### Quantitative polymerase chain reaction (qPCR) assay

Total RNA was extracted from cells using the TRI Reagent protocol (Sigma-Aldrich, Dorset, UK). A reverse transcription (RT) PCR kit converted 0.5 or 1 μg of RNA into complementary DNA according to the manufacturer’s instructions (nanoScript 2 Reverse Transcription Kit, primer design, Southampton, UK). Polymerase chain reaction primers were designed by Primer3 (HIN) as follows: 1) IFNγ sense, 5’-TGTCGCCAGCAGCTAAAACA-3’; antisense, 5’-ACTGAACCTGACCGTACATGCAGGCAGGACAA-CCATTA-3’; 2) IDO1 sense, 5’-AAAAGGATCCTAATAAG-CCCC-3’; antisense, 5’-ACTGAACCTGACCGTACACAGTCTCCATCACGAAATGA-3’; 3) IDO2 sense, 5’- GAGCTGCGGAGCTATCACAT-3’; antisense, 5’- ACTGAACCTGACCGTACACCACGTGGGTGAAGGATTGA-3’; 4) TDO sense, 5’- CCAGGTGCCTTTTCAGTTGC-3’; antisense, 5’-ACTGAACCTGACCGTACACTTCGGTATCCAGTGTCGGG-3’. The underlined sequence in the reverse primers was the additional Z sequence, which is complementary to the universal Z probe (TCS Biologicals Ltd., Oxford, UK). cDNA was diluted 1:8 and qPCR was performed using the Step One Plus RT-PCR mix (Applied Biosystems, Life Technologies Ltd, UK).

### Western blot analysis

Cells were rinsed with PBS and lysed in ice-cold lysis buffer containing a cocktail of protease inhibitors (Sigma, St. Louis, MO) and phosphatase inhibitors (Roche Applied Science, Indianapolis, IN) for 30 minutes. After lysates had been centrifuged for 15 minutes at 15000×g at 4°C, the supernatants were collected and their total protein concentrations were measured by the MicroBCA reagent (Pierce, Rockford, IL). Western blot analysis was performed after sodium dodecyl sulfate-polyacrylamide gel electrophoresis (equal aliquot of total proteins/lane) and transfer onto membranes. The proteins were hybridized with primary antibodies and the membranes were incubated with horseradish peroxidase (HRP)-conjugated secondary antibodies, which were subjected to the Amersham ECL system (GE, Trevose, PA) before visualizing signals with B:Box Chemi XX6 (Syngene, Cambridge, UK).

### ELISA analysis

We treated HMEVCa-D cells with MDA-MB-231 conditioned medium for 48 hours. The medium was centrifuged at 200×g for 15 minutes and the supernatant was collected for measuring TSP1 concentrations and Kynurenine concentrations. Each sample was measured in duplicate in each sample using TSP1 ELISA kit (Abcam, MA, USA), or L-Kynurenine ELISA kit (Immundiagnostik AG, Bensheim, Germany) according to the manufacturers’ instructions. The results were expressed as TSP1 (ng/ml) and L-Kynurenine (μM), respectively.

### IDO1 siRNA knockdowns IDO1 expression in ECs

IDO1 (INDO) ON-TARGET plus SMART-pool siRNA and ON-TARGET plus siCONTROL was purchased from Dharmacon RNA Technologies (Lafayette, CO, USA) as the following sequences:

Human INDO (NM_002164), sense, 5′-UCA-CCAAAUCCACGAUCAUUU-3′, antisense, 5′-PU-AUGCGAAGAACACUGAAAUU-3′; sense, 5′-UU-UCAGUGUUCUUCGCAUAUU-3′, antisense, 5′-PUAUGCGAAGAACACUGAAAUU-3′; sense, 5′-GUAUGAAGGGUUCU GGGAAUU -3′, antisense, 5′-PUUCCCAGAACCCUUCAUACUU-3′; sense, 5′-GAA CGGGACACUUUGCUAAUU-3′, antisense, 5′-PUUAGCAAAGUGUCCCGUUCUU-3′.

Log-phase HMVECa-D cells were seeded onto 6-well plates at the density of 200,000 cells/well. The cells were treated with IFNγ (10 ng/mL) and incubated at 37°C for 16 hours. 5 μl of siRNA or ON-TARGETplus siCONTROL Non-targeting Pool (20μM, Dharmacon) was added to 100 μl of serum-free DMEM and mixed. The mixture was added with 5 μl of Lipofectamine 2000 (Thermo Fisher Scientific, Waltham, MA, USA) at room temperature for 30 minutes before adding extra 800 μl of serum-free DMEM. Then the mixture was added to the cells. 1 mL of EGM with endothelial supplements was added to each well after 6 hours. Medium was changed after 24 hours. After 48 hours, kynurenine content of the medium was measured by ELISA. Harvesting of RNA extraction was followed by QRT-PCR analysis.

### Statistical analysis

We repeated all experiments at least three times. The statistical significance for the tissue microarray analyses was calculated by ANOVA. Overall survival was examined using Kaplan-Meier survival cures with Log-rank (Mantel-Cox) test. Also, ANOVA was used to calculate the difference among multiple groups of *in vitro* experiments. The significance between *in vitro* experimental and control groups was determined by Student’s *t*-test. The overall difference in IFNγ or IDO1 expression at the mRNA level using quantitative PCR was determined by Wilcoxon-Mann-Whitney analyses. Results are expressed as mean±SEM. Statistical analysis was performed using GraphPad Prism (version 6; GraphPad Software, Inc.) with p<0.05 considered statistically significant.

## SUPPLEMENTARY MATERIALS FIGURES AND TABLE


