# Supplementary material for: Association of breast carcinoma growth with a non-canonical axis of IFNγ/IDO1/TSP1
Source: Oncotarget. 2017 Jun 28;8(49):85024–39. doi: 10.18632/oncotarget.18781 (PMC5689591; doi:10.18632/oncotarget.18781)
Supplement: Supplementary file 1 [file oncotarget-08-85024-s001.pdf]

Association of breast carcinoma growth with a non-canonical axis of IFNγ/IDO1/TSP1

SUPPLEMENTARY MATERIALS

Supplementary Table 1: The criteria for histologic grade of breast cancer

	Glandular/tubular structures	Nuclear pleomorphism	Mitotic count (10 high power fields)
**Score 1**	>75%	Small nuclei, regular outline, uniform chromatin, little variant in cell size	≥7 mitoses/
**Score 2**	10% to 75%	Open vesicular nuclei, visible nucleoli, moderate variability in both size and shape	8-14 mitoses
**Score 3**	<10%	Vesicular nuclei, prominent nucleoli, marked variation in size and shape	≥15 mitoese

Grade 1: scores of 3,4,5
Grade 2: scores of 6 or 7
Grade 3: scores of 8 or 9

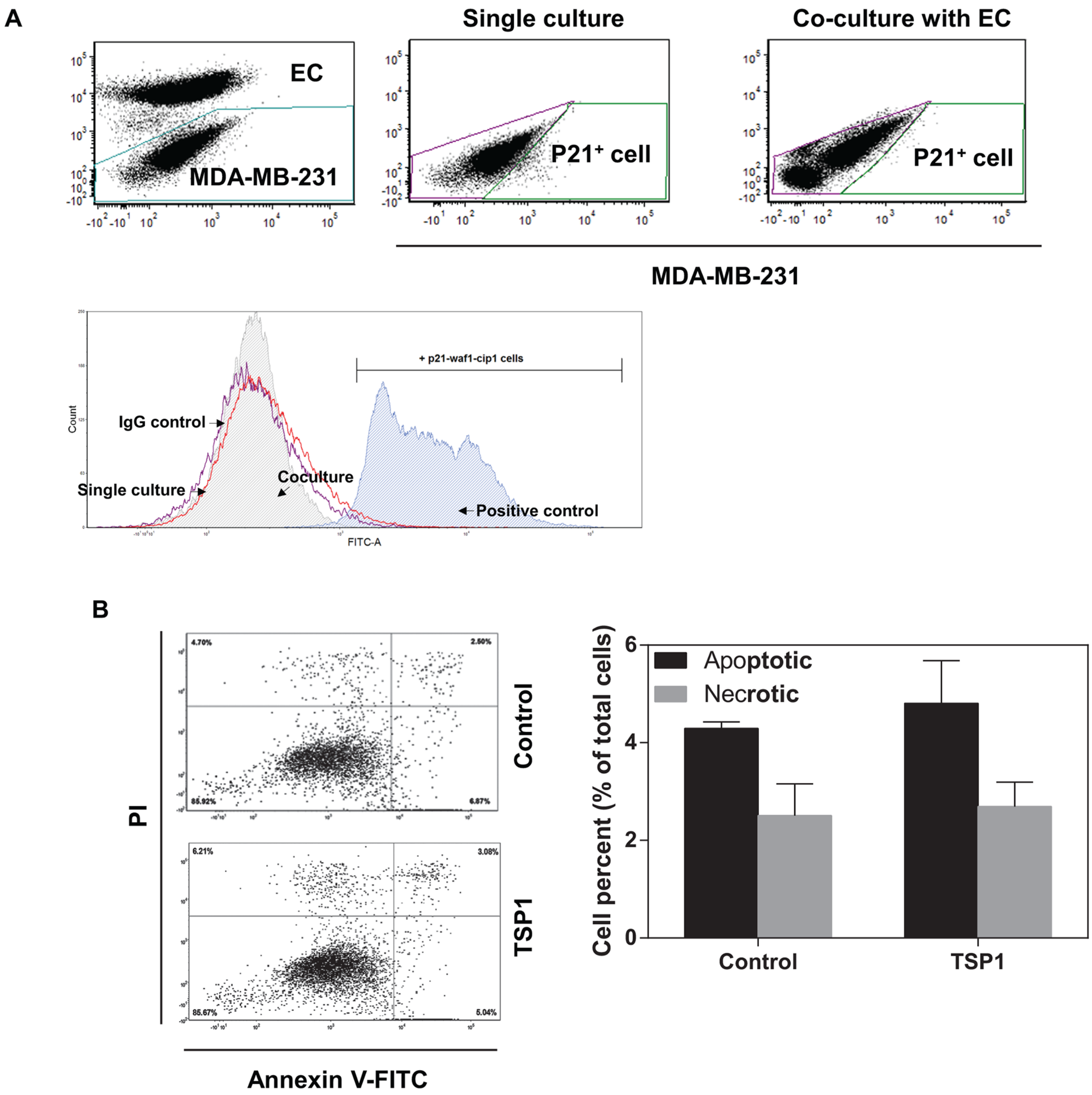

Supplementary Figure 1: (A) IDC cells and ECs were isolated from the co-culture through a cell sorting. (B) Apoptosis status of IDC cells treated with TSP1 was determined by flow cytometric analysis of Annexin-V/propidium expression.

A

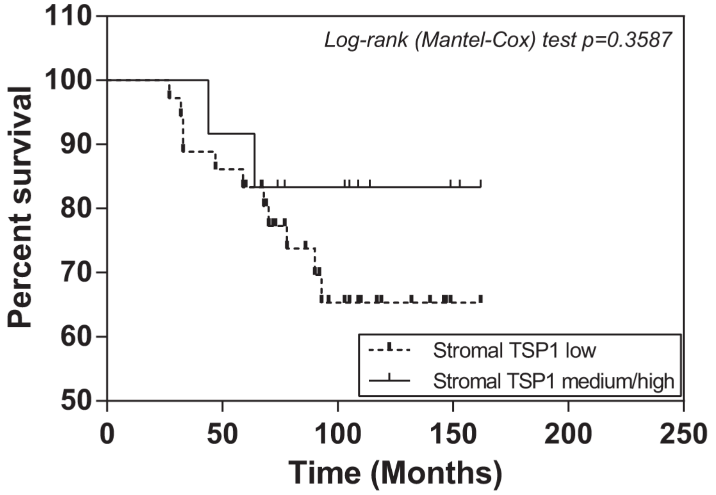

B

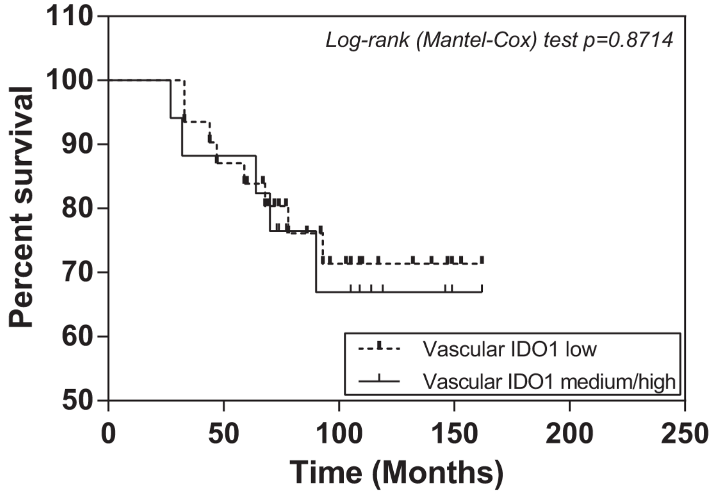

Supplementary Figure 2: (A) Kaplan-Meier IDC patient's survival curves stratified by two groups of histomorphometric scores of the stromal TSP1 (p=0.3587). **(B)** Kaplan-Meier IDC patient's survival curves stratified by two groups of histomorphometric scores the vascular IDO1 (p=0.8714).

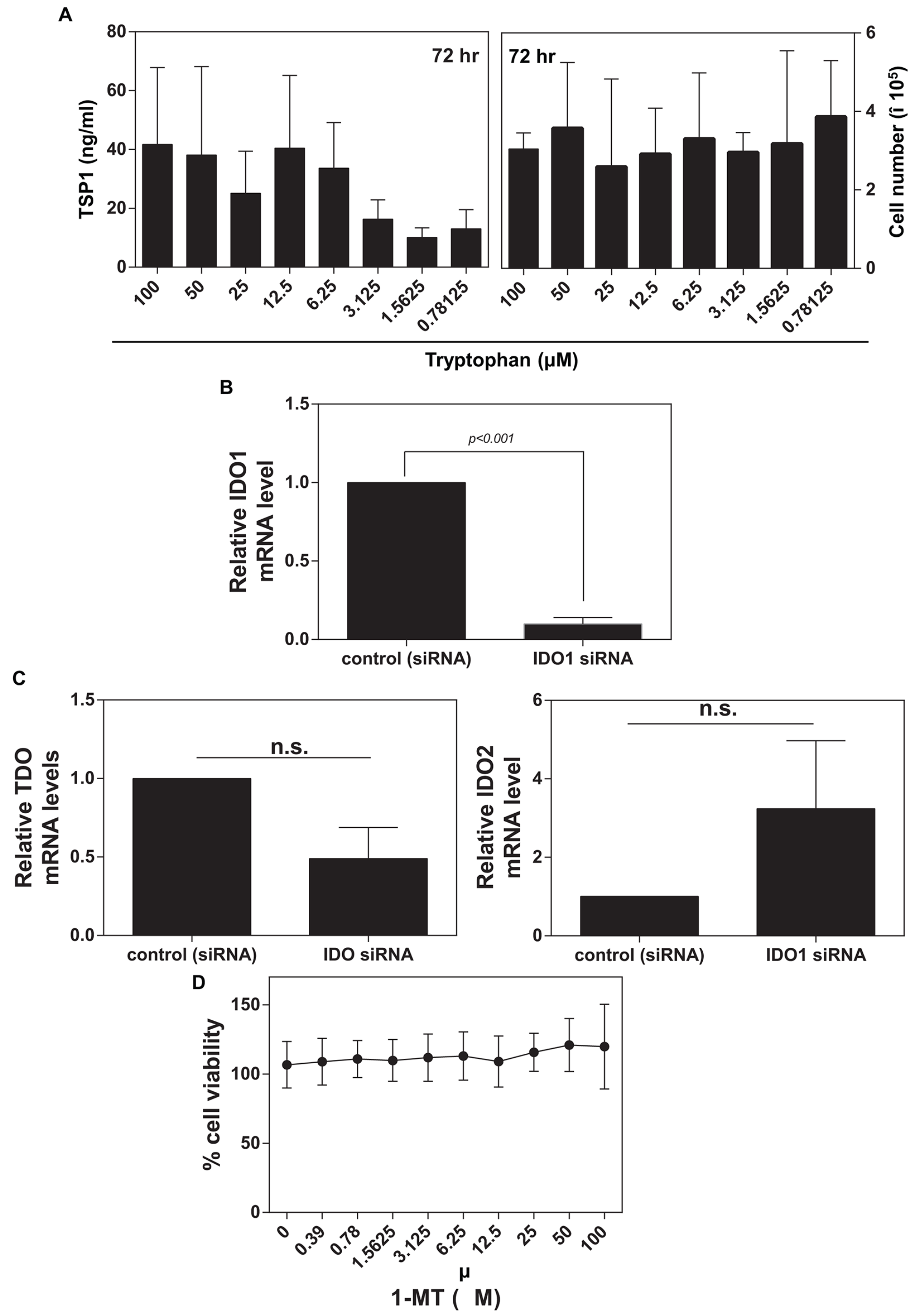

Supplementary Figure 3: (A) TSP1 concentrations was determined by ELISA analysis, indicating that tryptophan concentrations affect TSP1 secretion by ECs. (B) IDI siRNA significantly knockdowns IDO1 expression by ECs pre-treated with low glucose of MDA-MB-231 cell-conditioned medium. **(C)** The relative mRNAs of TDO and IDO2 was determined by QRT-PCR, showing that IDO1 knockdown did not cause detectable change in either of gene expression in ECs. **(D)** 1-methyl-tryptophan (1MT) does not change the cell viability up to 100 µM concentration.
